# Effect of Technological Treatments on Human-Like Leptin Level in Bovine Milk for Human Consumption

**DOI:** 10.3390/foods3030433

**Published:** 2014-07-23

**Authors:** Damiano Magistrelli, Fabia Rosi

**Affiliations:** Department of Agricultural and Environmental Sciences, University of Milan, via G. Celoria 2, 20133 Milan, Italy; E-Mail: fabia.rosi@unimi.it

**Keywords:** leptin, human-like immunoreactivity, bovine milk, commercial milk, infant formula, radio-immune assay, UHT, pasteurization, skimming

## Abstract

In this experiment, raw milk and commercially available full-cream UHT milk, semi-skimmed UHT milk, skimmed UHT milk, full-cream pasteurized milk, semi-skimmed pasteurized milk and infant formulas for babies between 6 and 12 months of age were analyzed by RIA, with a method using an antibody directed against human leptin and human leptin as reference standard. Raw milk and full-cream UHT milk did not differ for human-like leptin. Leptin content of full-cream pasteurized milk was not different to that of full-cream UHT milk, but it was 14% lower (*p* < 0.05) than that observed in raw milk. Human-like leptin level of semi-skimmed UHT milk was not different to that of semi-skimmed pasteurized milk, but it was 30% lower (*p* < 0.0001) than those of full-cream UHT and full-cream pasteurized milks. In skimmed UHT milk, leptin was 40% lower (*p* < 0.0001) than in full-cream UHT milk. Leptin was correlated (*p* < 0.001) with lipid content. Leptin level of infant formulas was not different to that of skimmed milks. Results suggest that the heat treatment (pasteurization or UHT) is not a modifier of human-like leptin content of edible commercial bovine milks, whereas the skimming process significantly reduces milk leptin level.

## 1. Introduction

Leptin, the product of the ob gene, is a 167-amino acids hormone characterized by a high degree of homology among species. It is mainly synthesized by adipose cells as an indicator of energy stores in the body [1]. Leptin enters the central nervous system by receptor-mediated transcytosis across the blood-brain barrier and targets the hypothalamus, the major effector organ for energy homeostasis, decreasing appetite and promoting basal metabolism, thereby reducing body fat mass [1]. The regulation of food intake and body weight is not the unique action of leptin in mammals. Leptin seems to be necessary for reproduction: circulating leptin levels increase at the onset of puberty [2], informing the central nervous system of the adequacy of energy stores for reproduction [1]. Leptin acts as permissive signal for the development of the sexual maturity [2] and stimulates GnRH release by the hypothalamus [3]. Moreover, leptin directly promotes gonadotropin secretion by interaction with specific receptors on the pituitary gland [3].

Leptin is also synthesized by extra-adipose tissues including placenta and gastric fundic mucosa [1]. In 1997, Casabiell *et al.* [4] and Houseknecht *et al.* [5] reported about the presence of leptin in human milk. Casabiell *et al.* [4] also suggested that mammary epithelial cells transfer leptin from blood into milk via a receptor-mediated process. One year later, Smith-Kirwin *et al.* clearly demonstrated that, in addition to leptin transfer from blood, mammary epithelial cells synthesize leptin [6], and both mechanisms account for the presence of leptin in milk [6,7]. To date, leptin has been detected in the milk of many species [7,8,9,10,11,12]. Milk leptin may act on the suckling’s physiology before and after its absorption into the bloodstream. Before its absorption, leptin may modulate gastrointestinal functions of the neonate, reducing its production by the gastric mucosa, possibly through a feedback mechanism [13], and regulating the development of the small intestine [14]. After its absorption into the neonate’s bloodstream, leptin modulates cytokine production, thymus size, activation of monocytes/macrophages, proliferation/apoptosis of T lymphocytes, thermogenesis and body growth [7]. Moreover, absorbed leptin regulates food intake, providing moderate protection to infants from an excess of weight gain [13,15].

The intake of milk leptin during the suckling period is thought to improve leptin and insulin sensitivity in adulthood, reducing the risk of obesity and related health complications, such as cardiovascular diseases and diabetes [16].

Highly efficient absorption of ingested leptin has been reported in rats until 9 days of age [7,13] and in neonatal pigs [7]. This absorption is mediated by receptors, but it may be enhanced by the immaturity of the neonatal gastrointestinal mucosa, which is permeable to macromolecules, as reported for other colostral proteins [7]. Leptin absorption capacity decreases according to the maturation of the gastrointestinal mucosa, and it is supposed to cease at weaning [13]. Anyway, the presence of leptin receptors on the gastrointestinal mucosa is not limited to the suckling period. Some authors report that chief and parietal cells of the stomach of adult humans and rats express both leptin and leptin receptors [17,18]. These species express leptin receptors also in the brush border, basolateral membrane, and cytoplasm of the enterocytes [19]. In adult rats, leptin is secreted into the gastric juice and reaches the duodenum in a biologically intact active form. Here, luminal leptin reacts with its intestinal receptor and may influence nutrient absorption, mucosal renewal and intestinal immunity, therefore participating in gut homeostasis [18,20]. Once gastric leptin has played its role on the enterocyte, one may expect degradation. By contrast, lumen leptin is internalized by the intestinal epithelium, transcytosed and delivered to blood and may potentially play important endocrine functions [20]. This latter result raises the challenging hypothesis that ingested human-like leptin could interact with its receptor on the gastrointestinal mucosa of the human consumer and potentially play a functional role. Humans consume a considerable amount of bovine milk, especially during infancy and childhood, but, contrarily to all the other mammals, milk intake is extended throughout the lifetime. This fact is not related only to the organoleptic characteristics of bovine milk, but is fostered by recommendations from nutritionists and sanitary authorities encouraging to drink milk because of its beneficial effects for calcium uptake and bone mineralization and as a source of valuable proteins [21]. The presence of leptin in bovine edible commercial milks and infant formulas, which are the major vectors of exogenous leptin in human nutrition, has been investigated over a decade ago [22], but without considering the effect of the technological treatments. Moreover, some authors have expressed doubts on the presence and identification of leptin in infant formulas [23]. According to this, the present study aimed at verifying human-like leptin immunoreactivity in raw and commercial bovine milks and infant formulas, in order to obtain information on the effect of technological treatments on milk leptin content.

## 2. Experimental Section

### 2.1. Samples and Analyses

During the first week of March, we collected 20 samples of raw milk from 10 different bovine farms of Lombardy, in the north of Italy. Raw milk was analyzed for composition by near infrared spectroscopy (NIRS).

We selected six brands of commercial milk (Parmalat^®^, Granarolo^®^, Carnini^®^, Centrale del latte di Milano^®^ Centrale del latte di Brescia^®^ and Auchan^®^) and collected two samples of full-cream ultra-high-temperature treated (UHT) milk, semi-skimmed UHT milk, skimmed UHT milk, full-cream pasteurized milk and semi-skimmed pasteurized milk, for each brand. During pasteurization and UHT treatments, milk is kept at 72 °C for 15 s or 135–140 °C for 1–4 s, respectively, according with the current legislation (D.P.R. 54/97) [24]. Milk skimming consists of a centrifugal separation of cream from raw milk. Generally, a portion of the separated cream is added back to milk to obtain a product with a standardized fat content [25].

We selected six infant formulas for artificial lactation of babies between 6 and 12 months of age (Mellin 2^®^, Plasmon Transilat 2^®^, Coop Crescendo 2^®^, Milupa Aptamil 2^®^, Nestlè Nidina 2^®^ and Humana 2^®^), reconstitute them according with the producer guidelines and collected two samples for each brand. All the selected infant formulas contained powdered bovine skimmed milk, which is obtained by a gentle removal of water (low temperature and low pressure) from skimmed milk using a spray-dry method, which coarsely consists of atomization of skimmed milk into fine droplets in a drying chamber in a flow of hot air, using a spinning disk atomizer or a series of high pressure nozzles. In the drying chamber, water evaporates, leaving a fine powder of around 6% moisture content, which is then subjected to processes of standardization and is added with natural surfactants or wetting agents and emulsifiers, to favor its dispersion into water [25].

Two milliliters of each sample were transferred in a 5 mL tube and sonicated by an ultrasonic probe for 3 min at 45 kHz on ice. Each sample was then defatted by addiction of 0.5 mL of chloroform (ClCH_3_), shaking on a vortex mixer for 1 min at 45 Hz and centrifugation at 4000× *g* for 30 min at 4 °C. Fats were removed since they could interfere with the leptin assay [26]. Supernatants were analyzed for leptin by radio-immune assay (RIA) with a method using an antibody directed against human leptin and human leptin as reference standard (Multispecies Leptin RIA kit-Millipore Corp., St. Charles, MO, USA), in order to measure human-like leptin immunoreactivity. The protocol was validated for bovine milk by: (1) parallelism test: serial dilutions of milk in assay buffer produced curves parallel to the standard curve, and (2) recovery test: known amounts of standard solutions added to milk produced proportional increases of leptin level. The limit of sensitivity of the test was 0.5 ng/mL, the inter- and intra-assay coefficients of variation were 8.7% and 3.6%, respectively.

### 2.2. Statistical Analysis

Data were analyzed by the ANOVA test, using SAS software version 8.01 (SAS Institute Inc., Cary, NC, USA). Differences between groups were determined by Student’s *t*-test. Results are presented as mean value ± SD. Milk leptin concentration is expressed as ng/mL of human equivalent (HE) leptin. Leptin content of raw milk was correlated with lipid, protein and carbohydrate content determined by NIRS, using a simple regression test. Similarly, leptin content of commercial milks (excluding the infant formulas) was correlated with the respective lipid, protein and carbohydrate content displayed on the package. Correlation analysis was not performed for infant formulas, since lipid content of formulas is mainly constituted by vegetal oils, so an eventual correlation between leptin (of animal origin) and lipid (of vegetal origin) content would be random.

## 3. Results

**Figure 1:**
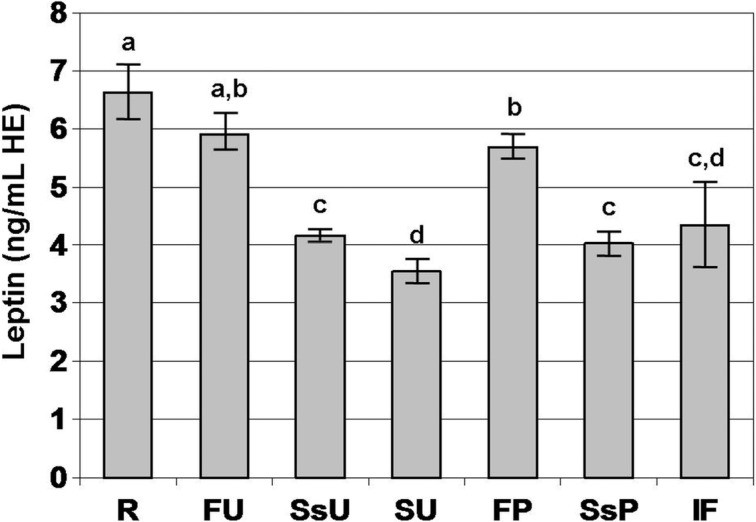
Mean levels of leptin in raw milk (R), full-cream UHT milk (FU), semi-skimmed UHT milk (SsU), skimmed UHT milk (SU), full-cream pasteurized milk (FP), semi-skimmed pasteurized milk (SsP) and infant formula (IF). Data are expressed as human equivalent (HE). Bars indicate SD. Histograms having unlike superscripts are statistically different.

The mean value and SD of each type of milk are presented in Figure 1. Human-like immunoreactive leptin was detected in all the analyzed milk samples. The highest level of leptin was observed in raw milk, the lowest one in skimmed UHT milk. Raw milk and full-cream UHT milk did not differ for human-like leptin level. No difference was observed between full-cream UHT milk and full-cream pasteurized milk, but human-like leptin content of full-cream pasteurized milk was 14% lower (*p* < 0.05) than that observed in raw milk. Human-like leptin level did not differ between semi-skimmed UHT milk and semi-skimmed pasteurized milk, but it was 30% lower (*p* < 0.0001) than that observed in full-cream UHT milk and full-cream pasteurized milk. In skimmed UHT milk, leptin content was 40% lower (*p* < 0.0001) than that observed in full-cream UHT milk.

In infant formulas, the level of human-like immunoreactive leptin ranged between 2.01 ng/mL (Milupa Aptamil 2^®^) and 5.76 ng/mL (Mellin 2^®^), showing a great variability among brands (Figure 1). In infant formulas, mean level of leptin was not different to that observed in semi-skimmed and skimmed milks.

**Figure 2:**
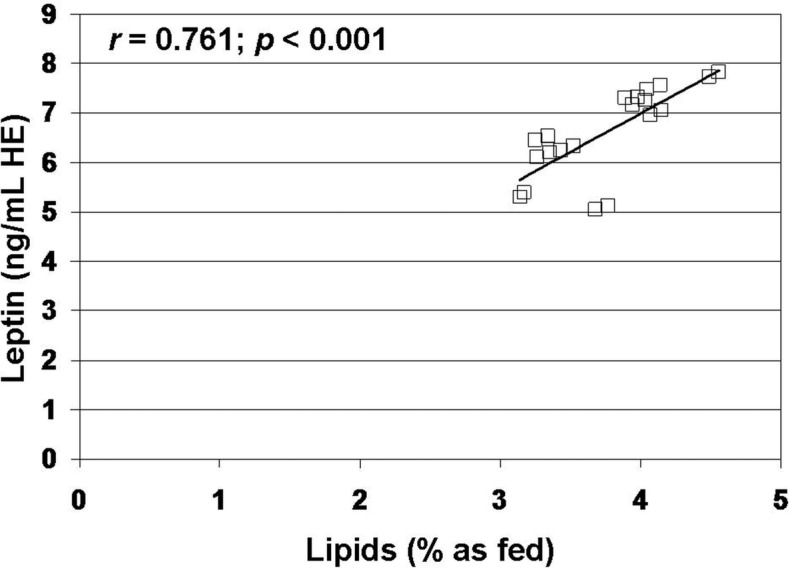
Analysis of correlation between leptin and lipid content of raw milk. Data on leptin are expressed as human equivalent (HE).

**Figure 3:**
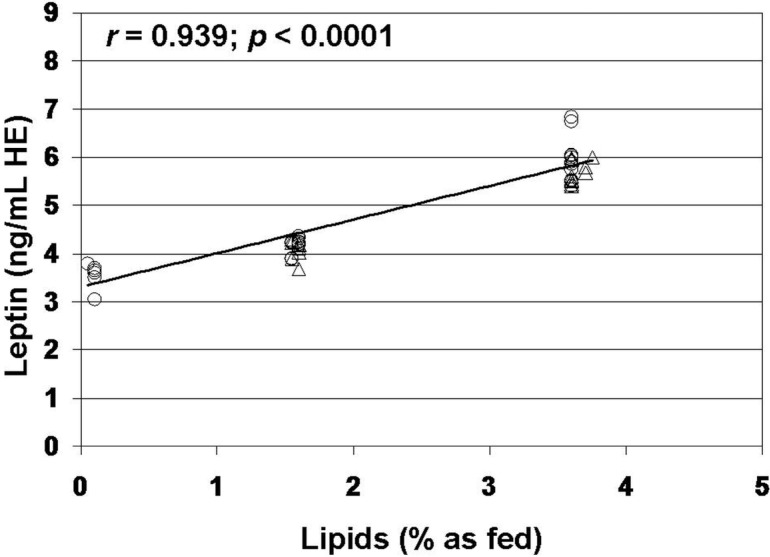
Analysis of correlation between leptin and lipid content of commercial milks. Data on leptin are expressed as human equivalent (HE). Circles are used for UHT milk samples. Triangles are used for pasteurized milk samples.

The regression test showed a strong correlation between leptin and lipid content (*r* = 0.761, *n* = 20, *p* < 0.001) in raw milk (Figure 2). In raw milk, leptin content did not correlate with protein (*r* = −0.225, *n* = 20, *p* > 0.10) and carbohydrate content (*r* = −0.014, *n* = 20, *p* > 0.10). Similarly, in commercial milks, leptin content was correlated with lipid content (*r* = 0.939, *n* = 60, *p* < 0.0001) (Figure 3), but it was not correlated with protein (*r* = −0.125, *n* = 60, *p* > 0.10) and carbohydrate content (*r* = −0.044, *n* = 60, *p* > 0.10).

## 4. Discussion

Since bovine leptin shares approximately 87% homology with human leptin [27], one could wonder if the leptin present in bovine edible milks can interact with leptin receptors on the gastrointestinal mucosa of the human consumer. To shed light on this topic, we measured the amount of leptin in bovine milks for human consumption, available on the markets of northern Italy, with a method using an antibody directed against human leptin and human leptin as reference standard. The study shows a limitation in the number of samples, collected in one single period of the year. This limitation can be justified by the fact that, in modern farms (as those selected for this trial), cows are able to breed year round. This, together with climate control systems, helps in minimizing seasonal variations in milk production and composition [28]. Moreover, before marketing, milk is subjected to processes of standardization in order to provide the consumer with a regular product, throughout the year [25,28].

Due to the species-specificity of peptidic hormones and the risk of poor cross-reactivity [29,30], some authors set up a method specific for bovines [29] and ovines [30]. Comparison between results obtained on ruminants with a method specific for human leptin and results obtained on ruminants with species-specific methods showed that, although the results obtained with the method specific for human leptin were 20%–50% lower than those obtained with the specific methods, they were highly correlated (*p* < 0.001) [29,30,31].

The highest level of leptin was observed in raw milk, the lowest one in skimmed UHT milk. Human-like leptin content of raw milk was not different to that of full-cream UHT milk, but it was significantly higher than that observed in full-cream pasteurized milk. This result may indicate that UHT treatment does not have any effect on the tri-dimensional structure of leptin, whereas pasteurization could modify leptin, reducing its detectable level in milk. However, no difference was observed between UHT milks and pasteurized milks, suggesting that heat treatment (pasteurization or UHT) is not a selective factor for leptin content in edible bovine milks, available on the markets of northern Italy.

Our results showed a progressive reduction of leptin content with the increase of intensity of the skimming process. Moreover, the content of leptin was strongly correlated with milk lipid content, as previously observed [9,22]. A major fraction of milk leptin is associated with milk fats [7,9]. In goat milk, about 60% of total leptin is associated to the fat fraction [32]. In mare milk, the concentration of leptin in the fat fraction is 30 times higher than that of skimmed milk [8]. It is clear, therefore, that milk skimming reduces milk leptin content. In humans, milk skimming reduces by more than 70 times the content of leptin [6]. According to this, some authors advanced the hypothesis that leptin is secreted by the mammary gland within the milk fat globules or associated to milk fat globule membranes, whereas leptin content of the skimmed fraction (milk serum) derives from the systemic circulation [6].

Human-like leptin was detected in all the selected infant formulas, in concentrations lower than those measured by Lage *et al*. [22], who observed that leptin level was higher in infant formulas than in full-cream milk. O’Connor *et al*. [33] affirm that RIA leptin methodology causes an overestimation of leptin in infant formulas, possibly due to interference with supplemented iron and emulsifiers, among other additives. As reported by the manufacturers, the most used emulsifiers in infant formulas are mono-, diglygerides and soy lecithin (phosphatidylcholine), which are all soluble into chloroform. In the present experiment, addition of chloroform to sonicated milk, before leptin analysis, avoided interference by emulsifiers. Resto *et al*. [34] extracted proteins from infant formulas by western blotting, in order to avoid interferences, and analyzed them for leptin by RIA, founding no detectable leptin in all the analyzed formulas. The authors hypothesized that infant formulas do not contain leptin because whey proteins added to formulas are isolated from skimmed bovine milk, and leptin associated with milk fat globules are removed during the skimming process [34]. This inconsistency among results from different studies could be explained by differences in the composition of infant formulas. According to the *Codex Alimentarius*, infant formulas are breast-milk substitutes for nutrition of infants, *i.e.*, persons under 1 year of age (CODEX STAN 72). Infant formulas distributed in the Italian markets are divided into “starter”, for artificial nutrition of babies between 0 and 6 months of age, and “follow-on”, for artificial nutrition of babies between 6 and 12 months of age. Some starter infant formulas do not contain milk but whey protein isolated from skimmed milk (e.g., Nestlè Nidina 1^®^). Some others contain powdered skimmed milk (e.g., Mellin AR1^®^ and Milupa Aptamil AR1^®^). As reported by the manufacturers, all the infant formulas analyzed in the present study contain powdered bovine skimmed milk, which is obtained by a spray-dry method based on evaporation of water under low temperatures and low pressures, for few time, in order to maintain the nutritional value of milk [25], with the exception of a very small percentage of lactose (about 1%), which can isomerize into lactulose [35]. It is likely, therefore, that leptin is not denatured by the spray-dry method, and powdered skimmed milk constitute a vector of immunoreactive leptin in infant formulas. Other authors observed that sterilization by autoclaving milk at a constant temperature of 100 °C for 5 min significantly decreased detectable leptin levels, because of leptin denaturation [34], but this latter treatment is certainly more intense than spray-dry. The sterilizing process just described, in fact, has been shown to alter the natural properties of milk, reducing milk fats (*p* < 0.01) and proteins (*p* = 0.14) [34].

## 5. Conclusions

The heat treatment (pasteurization or UHT) is not a modifier of human-like leptin content in edible commercial bovine milks, available on the markets of northern Italy. By contrast, human-like leptin level of milk is significantly reduced by the skimming process, since a major fraction of milk leptin is associated with milk fats. However, a minor fraction of milk leptin is present in the skimmed fraction of milk (milk serum). As consequence, human-like immunoreactive leptin is clearly detectable in infant formulas containing powdered skimmed milk, such as those for artificial lactation of babies between 6 and 12 months of age. It means that commercial bovine milk is a vector of human-like immunoreactive leptin in human nutrition, anyway, questions whether human-like milk leptin can maintain its biological activity after its intake by the human consumer and whether human-like leptin may interact with its gastrointestinal receptors and play a functional role are still to be answered.
